# Model Building in Forensic Psychiatry: A Machine Learning Approach to Screening Offender Patients with SSD

**DOI:** 10.3390/diagnostics12102509

**Published:** 2022-10-16

**Authors:** Lena Machetanz, David Huber, Steffen Lau, Johannes Kirchebner

**Affiliations:** Department of Forensic Psychiatry, Psychiatric Hospital, University of Zürich, 3002 Zurich, Switzerland

**Keywords:** model building, machine learning, artificial intelligence, schizophrenia, forensic psychiatry, adverse treatment course

## Abstract

Today’s extensive availability of medical data enables the development of predictive models, but this requires suitable statistical methods, such as machine learning (ML). Especially in forensic psychiatry, a complex and cost-intensive field with risk assessments and predictions of treatment outcomes as central tasks, there is a need for such predictive tools, for example, to anticipate complex treatment courses and to be able to offer appropriate therapy on an individualized basis. This study aimed to develop a first basic model for the anticipation of adverse treatment courses based on prior compulsory admission and/or conviction as simple and easily objectifiable parameters in offender patients with a schizophrenia spectrum disorder (SSD). With a balanced accuracy of 67% and an AUC of 0.72, gradient boosting proved to be the optimal ML algorithm. Antisocial behavior, physical violence against staff, rule breaking, hyperactivity, delusions of grandeur, fewer feelings of guilt, the need for compulsory isolation, cannabis abuse/dependence, a higher dose of antipsychotics (measured by the olanzapine half-life) and an unfavorable legal prognosis emerged as the ten most influential variables out of a dataset with 209 parameters. Our findings could demonstrate an example of the use of ML in the development of an easy-to-use predictive model based on few objectifiable factors.

## 1. Introduction

With the expansion of digitalization in healthcare and the increasingly standardized documentation of clinical courses, wide ranges of medical data are available today. More than ever before, scientists are therefore able to investigate complex issues and derive practicable models for use in everyday clinical practice. To do so, modern statistical approaches with the ability to handle a large quantity of data and variables are necessary. An example for such an advanced statistical approach is machine learning (ML), which allows (1) the analysis of a variety of variables and their interplay through the application of complex mathematical algorithms and (2) a quantification of the quality of the applied statistical model [1,2]. Already widely used in areas such as consumer analytics, model building using ML is also applied in various contemporary medical specialties, e.g., to predict treatment outcomes in oncology, to improve medical imaging, and, very recently, in the prevention and detection of COVID-19 [3,4,5]. The application of advanced statistics also opens new possibilities in the field of psychiatry, especially because many psychiatric phenomena are not monocausal in their origin but develop from a set of conditions of various factors [6,7].

In forensic psychiatric care of patients with a schizophrenia spectrum disorder (SSD), treatment courses are often lengthy, highly cost-intensive due to the need for higher security measures than in general psychiatry, and challenging due to involuntariness of the hospitalization and treatment [8,9,10,11]. While the demand for forensic psychiatric treatment capacities is ever-growing, the difficulty to generate appropriate resources, e.g., trained nursing staff or doctors, remains [12,13,14]. The justice system and, with it, the treatment of offender patients is financed through taxation; therefore, decision on the allocation of resources can be politically and socially controversial associety’s need for security is often set against the desire for a risk management that is as cost-efficient as possible [15,16]. Although it seems obvious that patients with SSD and a history of violent or disorderly behavior are at high risk for recurrence of such behavior, the course of inpatient court-mandated therapy is quite heterogeneous within the patient population: Adverse treatment events, such as escape and absconding, physical aggression, self-harm or substance use only occur in a small percentage of all patients [6,17,18,19]. A scattershot approach in resource allocation is therefore inefficient at best and negligent at worst, because already-scarce resources are spread too thin, and caregivers may not be able to uphold the necessary treatment standards. Analogous to the risk assessment tools commonly used in forensic psychiatry to assess the risk of reoffending, for example the Historical Clinical and Risk Management 20 (HCR-20) or the Violence Risk Appraisal Guide (VRAG), there is thus a need for a tool to predict adverse treatment courses in the correctional system [20,21].

Using ML, the present study aimed to develop a first basic model for the anticipation of adverse treatment courses based on simple and easily objectifiable parameters in offender patients with a schizophrenia spectrum disorder (SSD). 

## 2. Materials and Methods

Our population consisted of 370 offender patients with a diagnosis of SSD according to the ICD-9 (295.x) and ICD-10 (F20-29.x), who had been in court-mandated treatment at the Center for Inpatient Forensic Therapies of the University Hospital of Psychiatry Zurich [22,23]. Data from their files were extracted retrospectively by a psychiatrist with +5 years of forensic experience according to a standardized rating protocol based on a set of criteria originally proposed by Seifert et al. [24,25,26]. To evaluate for inter-rater reliability, a second independent rater encoded a random subsample (10% of all cases). With a Cohen’s Kappa of 0.78, inter-rater reliability was considered substantial [27]. The extensive files consisted of professionally documented anamneses, psychiatric/psychologic inpatient and outpatient reports, police reports, testimonies, court proceedings, reports from social workers, and biannual extensive reports from clinicians as well as the nursing and care staff. The comprehensive dataset derived from the file contents contained items from the following domains: social-demographic data, childhood/youth experiences, psychiatric history, past criminal history, social/sexual functioning, details on the offence leading to the current forensic hospitalization, imprisonment, and particularities of the current hospitalization and psycho-pathological symptoms. For definition of the latter, an adapted positive and negative syndrome scale (PANSS), with symptoms being rated on a three- instead of a seven-tier scale, was used (completely absent, discretely present, substantially present) [28]. 

The dataset has already been evaluated in other studies as part of a larger, ongoing project with the goal of providing further knowledge about the complex field of offender patients with SSD [6,8,17,29,30,31,32,33,34,35,36,37]. Whereas the aforementioned studies by the authors have so far aimed to exploratively examine the complex interplay of various influential factors in different phenomena to provide a better understanding of them and have tested other outcome variables (e.g., self-harm), the present work is a first approach to develop a clinical model for the prediction of certain events during the hospitalization in question. While the same database serves as basis for several analyses covering many objectives in this field of research, and although there are a few overlapping parameters, it still contains a substantial number of unique variables, thus resulting in different theoretical and practical conclusions and implications. An overview of the basic demographics of the population is shown in Table 1. Further details on data collection regarding our population can be found in Lau et al. [33]. 

**Figure 1:**
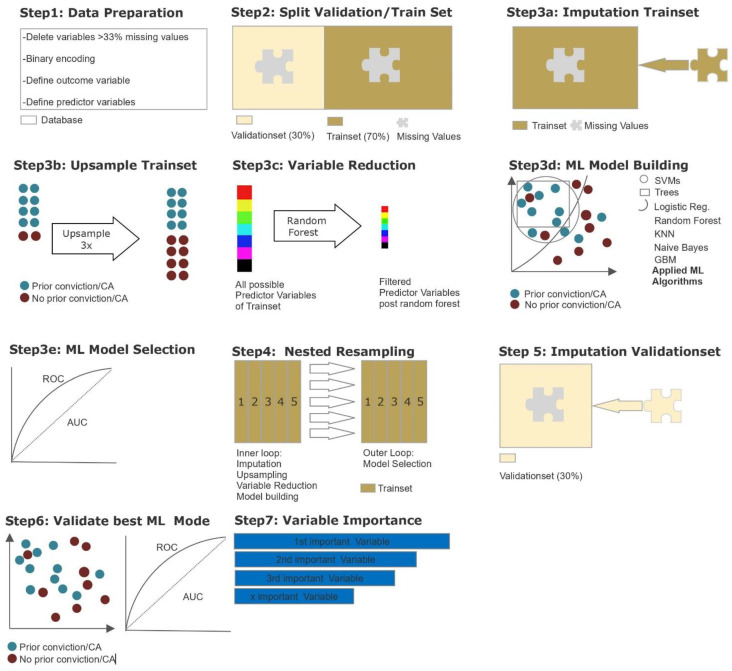
Statistical procedures using machine learning.

Parts of the following section were already published in another study from our research group and are partly replicated here due to the use of the same methodology [31]. To identify the most relevant variables out of a large quantity of parameters and the model providing the best predictive power, we used supervised ML due to explorative nature of this study. An overview of the statistical steps is shown in Figure 1 and is further described in detail below. All the steps were performed using R version 3.6.3. (R Project, Vienna, Austria) and the MLR package v2.171 (Bischl, Munich, Germany). CI calculations of the balanced accuracy were conducted using MATLAB R2019a (MATLAB and Statistics Toolbox Release 2012, The MathWorks, Inc., Natick, MA, USA) with the add-on “computing the posterior balanced accuracy” v1.0. All raw data were first processed for ML (see Figure 1, Step 1): Several categorical variables were converted to binary code. Continuous and ordinal variables were not adjusted. Due to the retrospective nature of the study and the large number of variables included, there were missing values among variables. This especially applied to information on the broader biographical history of patients, although forensic records were comprehensive. Variables with more than 33% missing values as well as parameters without reference to the forensic-psychiatric hospitalization (e.g., parameters regarding adolescence) were eliminated, leaving a set of 209 possible variables. The independent variable “*prior compulsory admission (CA) and/or conviction*” was defined as previous compulsory admission to a psychiatric hospital due to danger to others and/or self-endangerment and/or prior criminal convictions according to the patients’ criminal record, and was dichotomized into (a) “present” and (b) “not present”. After the elimination of cases with missing data, a total of 356 patients remained. With a total of 265 patients, the majority (74.4%) had had a prior conviction or compulsory admission (in Switzerland: FU/FFE), while 91 (25.6%) did not. The majority of patients were single at the time of the offence leading to the referenced forensic hospitalization and had been diagnosed with schizophrenia (F20.x acc. To ICD-10), while other diagnoses from the psychosis spectrum, for example schizoaffective disorder, were less prevalent (see Table 1).

After data preparation, the database was divided into one training and one validation subset (see Figure 1, Step 2). The training subset, including 70% of all cases, was used for variable reduction and model building/selection. To enable the flexible application of all ML algorithms, imputation of missing values was carried out by mean for continuous variables and by mode for categorical variables included in the MLR package, and imputation weights saved for later were reused on the validation subset (see Figure 1, Step 3a). As the outcome variable was unevenly distributed, a random up-sampling at a rate of 3 was conducted, leading to a more balanced outcome (see Figure 1, Step 3b). A major objective of the present study was to identify the most important variables from 209 possible variables. As a decrease in variables can counteract overfitting while maintaining computing times in initial model building at an acceptable level, we performed a variable reduction through random-ForestSRC, down to the point where the AUC did improve by no more than 5% through adding another item (see Figure 1, Step 3c). This led to a variable reduction down to the 10 most predictive variables. As the database was relatively small for ML purposes and our focus lay on variable extraction and prediction, we applied discriminative model building with logistic regression, trees, random forest, gradient boosting, KNN (k-nearest neighbor), support vector machines (SVM), and as an easily applicable generative model building, naïve Bayes (see Figure 1, Step 3d). Hyperparameter tuning was used to adjust the default functioning of algorithms in order to identify the most efficient model. The final hyperparameters applied in each model are provided in the Appendix A. 

For each model, performance was calculated and assessed in terms of its balanced accuracy (the average of true positive and true negative rate, better suited for model evaluation and calculation of confidence intervals in imbalanced data) and goodness of fit (measured with the receiver operating characteristic, balanced curve area under the curve method, ROC balanced AUC). Specificity, sensitivity, positive predictive value (PPV), and negative predictive value (NPV) were also evaluated. Our training dataset was artificially balanced; therefore, the model with the highest AUC was chosen for final model validation with the test subset (see Figure 1, Step 3e). Finally, to prevent overfitting, a nested resampling approach was employed. For this purpose, we used a nested resampling model with the inner loop performing imputation, oversampling, variable filtration, and model building within fivefold cross-validation, and the outer loop for performance evaluation also embedded in fivefold cross-validation (see Figure 1, Step 4). Through cross-validation, five different equal-sized subsamples of our dataset were artificially created, allowing one subset to serve as training set for our model, while the remaining four subsets allowed the evaluation of the accuracy of the learned model [38,39]. To evaluate the model selected before, we applied the validation subset, which included 30% of all cases (see Figure 1, Steps 5–7). The imputation weights previously stored were reused on the validation subset (see Figure 1, Step 5). Then, the selected model was applied for validation (see Figure 1, Step 6). The identified variables were finally ranked according to their indicative power (see Figure 1, Step 7).

## 3. Results

### 3.1. Model Calculation

Out of seven different models, gradient boosting was identified as the ML algorithm with the best performance measures (see Table 2). With a sensitivity of 69% and a specificity of 70%, the AUC yielded 0.76. 

Table 3 provides an overview over the distribution of the 10 variables identified as most predictive during nested resampling. These variables were then used in the final model.

The performance measures of the final gradient boosting model are shown in Table 4. As expected, due to the uneven outcome variable distribution in the validation set, the balanced accuracy of 67% and AUC of 0.72 were lower than in the initial training model (see Table 3, balanced accuracy: 69%, AUC 0.76), but still meaningful: With a sensitivity of 90%, patients with prior CA/conviction could be identified correctly on the basis of the 10 predictor variables in 9 out of 10 cases. Patients with no prior CA/convictions were identified correctly as such with lower reliability(specificity of 50%). Due to the high prevalence of prior CA/convictions in our population (74%), the negative predictive value (NPV) was much lower than the positive predictive value (PPV). Differences between the performance parameters regarding sensitivity and specificity and the PPV and NPV may result from the unbalanced population.

### 3.2. Predictive Variables Regarding Prior CA/Convictions

**Figure 2:**
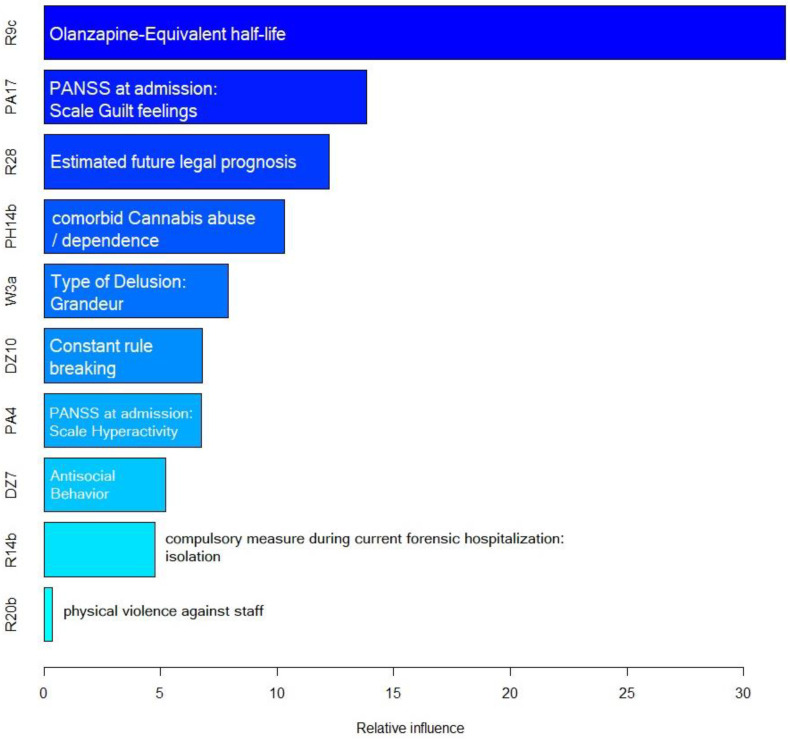
Variable importance Acc. to their relative influence in the final model.

The distribution of the importance of the predictive variables of the final validation model is demonstrated in Figure 2 as a one-sided tornado graph, ranked according to their relative influence.

*Olanzapine-equivalent half-life*, defined as the half-life (in hours) of the cumulative dose of antipsychotic monotherapy prescribed to the patient at admission converted to olanzapine-equivalents in milligrams, was identified as by far the most predictive variable. A group of variables with high predictive value in the final model was the group of adverse events during the current hospitalization: These included *rule breaking*, *antisocial behavior*, *isolation as compulsory measure*, and *physical violence against staff*. Another group of predictive variables regarded illness and symptomatology (*hyperactivity* and *guilt feelings* according upon admission according to the PANSS, *delusions of grandeur*, *cannabis abuse or dependence* as comorbidity). Lastly, the *estimated legal prognosis* at the time of discharge from the current forensic hospitalization, according to a forensic psychiatric evaluation, was identified as predictor variable as well. 

## 4. Discussion

The ability to correctly predict unfavorable or adverse events in medicine can improve health care in many domains: Taylor-made medical care complies with the individual needs of the patient, may help clinicians in risk stratification, allows economic resource allocation, and, finally, is more cost-efficient than a “one fits all” approach. A medical specialty, where these considerations are of great relevance due to a growing demand, shortage of trained staff and high costs, is the field of forensic psychiatry. Ideally, a screening tool predicting difficult treatment courses and adverse events allows psychiatrists to detect patients at risk and install appropriate interventions for prevention already upon admission of a new patient to forensic inpatient care. Such a tool, developed on a scientifically sound basis, can enable clinicians to provide a more personalized treatment approach, and might consequently be a valid juridical argument to differentiate in imposed penitentiary regimes. At the same time, there is usually little information regarding the patient upon admission from which such a screening could be conducted. This is even more so for patients with SSD, whose perception of reality is altered and whose cognitive functions are impaired by their underlying disorder, making it difficult to obtain a medical history. Therefore, it seems sensible to design a model based on easily accessible and objective parameters. 

When trying to build a predictive model from a quantity of covariates with complex interactions and higher-order terms among said covariates, generalized linear regressions are of limited use, as they require linearity and additivity to hold for the underlying data [40]. However, neither one nor the other underlies psychiatric illness and human behavior. Rather, psychiatry and psychology deal with multifactorial phenomena and complex sets of conditions. Here, the application of modern statistical methods, such as ML, can be of use. Thus far, the application of ML has been rare in the field of forensic psychiatry. Previous studies have mainly explored heterogenous forensic populations, e.g., for purposes of recidivism risk prediction, and have not focused on patients with SSD in particular [41,42]. The authors’ former publications, which evaluated a more homogenous population of offender patients with SSD exclusively, mainly focused on providing a better understanding of complex, multifactorial phenomena, such as stress, criminal recidivism, migration experience, self-harm, and aggressive behavior [6,18,30,32,37]. 

The study presented here aimed to provide a first exploratory model for screening adverse treatment events and complicating aspects in offender patients with SSD upon their admission to inpatient therapy, using prior compulsory admission and/or conviction as easily objectifiable and verifiable parameters. Out of seven different ML algorithms, gradient boosting emerged as the one with the best performance parameters. Gradient boosting is considered one of the strongest algorithms for building predictive models and has successfully been used in various predictive models in medical research, e. g. in the development of a sepsis screening tool or in identifying cardiovascular disease [1,40,43,44]. The idea behind gradient boosting is to create a strong composite model through combination of many simple models, so called “weak learners” [43]. This allows the evaluation of complex data structures from a large quantity of possible predictors. However, this also makes gradient boosting models prone to overfitting, thus possibly including data noise and outliers in the learned concept [44,45]. In our statistical analysis, we tried to counteract this phenomenon using a nested resampling approach as described in detail in the methodology. 

Out of over 209 variables, our findings defined ten factors most likely to be expected from patients with a history of CA and/or convictions. From a preventive point of view, the most valuable variables seem to be those concerning unfavorable events during further treatment. Such events that could be expected from patients with prior CA/conviction with a high likelihood were *rule breaking*, *antisocial behavior*, *physical violence against staff* and the *use of isolation as compulsory measure*. While the ladder cannot actually be considered an adverse event caused by the patient but is rather a consequence of aggressive and violent behavior, the other items are worth considering as they may be preventable with individual interventions. In general, there is an elevated prevalence of antisocial personality traits and behavior in patients with SSD [46,47]. However, it comes as no surprise that these variables emerged as even more frequent in SSD patients with prior CA/conviction: antisocial, violent and rule breaking in the past have been identified as risk factors for further similar behavior in both offender and non-offender patient populations [6,34,48,49]. A history of CA itself has already been identified as risk factor for violent inpatient behavior in non-offender populations [50]. 

Regarding symptoms to be expected upon admission, patients with prior CA/convictions showed *more hyperactivity* and *fewer guilt feelings* (according to the PANSS) as well as more *delusions of grandeur* than patients without prior CA/convictions. The latter is well explainable, as feelings of superiority and grandiosity may interfere with a patient’s willingness to comply to rules and follow orders, which could lead to an increased likelihood of this specific delusional content in the population with prior CA/conviction [51]. Decreased feelings of guilt seem to be a general finding in patients with SSD, as self-conscious emotions—in contrast to basic emotions—require higher-order cognitive processes, which are often heavily impaired in SSD [48]. The fact that this is even more so the case in patients with prior CA/conviction could, on one hand, be explained by a possible higher prevalence of antisocial personality traits amongst this subgroup. On the other hand, it can be hypothesized that patients who were either compulsorily admitted or have a history of unlawful behavior have more severe courses of their disorder and are therefore more severely affected in their cognitive functions, including the ability to process self-conscious emotions. Hyperactivity, or excitement, as form of disruptive behavior, has also been identified as being associated with endangerment of others in patients and consequently CA or convictions with SSD and other psychotic disorders in previous studies [49,52]. 

Patients with prior CA/convictions were also more likely to have *cannabis abuse or dependence* as a comorbidity. This is in line with current findings of another study with over 2000 participants, in which cannabis use was significantly associated with an increased likelihood of CA [53]. This is also true for a linkage between continuous cannabis use and violent behavior [54]. This has direct clinical implications regarding treatment: The comorbidity of substance use disorders in general is known to aggravate the course of disorder of offender patients with SSD in terms of severity of symptoms and expression of antisocial behavior [36,55]. Affected patients with the double diagnosis of SSD and cannabis abuse or dependence may likely benefit from appropriate treatment focusing on both to reduce criminal behavior and improve reintegration into society [56]. 

The variable with by far most influence on the model was the *olanzapine-equivalent half-life* at the time of admission as measure of the cumulative antipsychotic dose a patient received: Patients with a prior CA/conviction received a higher cumulative dose than patients with no prior CA/conviction. This may seem somewhat obvious, given the assumption that patients with the need for CA, as well as patients with prior criminal convictions, are more severely affected by their illness, and thus require a more extensive pharmacotherapy, e.g., in form of higher dosage or polypharmacy. 

Lastly, patients with prior CA/convictions received a more unfavorable *estimated legal prognosis* upon discharge from the forensic-psychiatric hospitalization. This is not surprising, as forensic-psychiatric risk assessment includes not only dynamic, but also static factors, which, if present, can negatively influence the legal prognosis regardless of the patient’s state at discharge. Previous convictions and previous endangerment of others are known risk factors for reoffending in patients with SSD, and, therefore, will likely be considered when evaluating the legal prognosis [57]. The item is also consistent with other two factors influencing our model, which are known to have a negative impact on criminal recidivism: substance use and antisocial behavior [57,58,59,60]. 

In summation, when looking at a patient with a history of CA and or criminal conviction, our results seem to paint a picture of a rather agitated patient with delusions of grandeur as expression of their psychosis, antisocial, even violent behavior, and a comorbid cannabis use disorder, who consequently has an increased need for antipsychotics and compulsory measures, and is assigned an unfavorable legal prognosis upon discharge. 

A major limitation of our study is that the analysis was based solemnly on retrospectively collected data. While data extraction followed a standardized protocol, this approach cannot provide the same data quality as data collection from a prospective longitudinal sample. This especially applies to variables with a certain subjective component, where the evaluation of the patients’ perspective through the researchers would have been valuable, e.g., patients’ stress level. While this may be partially counteracted through the substantial inter-rater reliability, possible bias effects remain. Another limitation is our sample size of *n* = 356, which, while rather large regarding the subspecialty of forensic psychiatry, can hardly be considered “big data” when compared with datasets of other medical specialties using ML algorithms. Therefore, to draw robust causal conclusions, a reproduction of the study as prospective design with a larger patient population is advised. While the performance parameters of the final model could show its ability to successfully identify clinically relevant variables to be expected from patients with prior CA/conviction, one must be mindful of the ethical implications of such a predictive model. The already vulnerable population of offenders with severe mental illnesses may be exposed to even more stigmatization after being labeled a “problematic patient” already upon admission. Finally, while the majority of offender patients still are men, the fact that our sample included fewer than 10% female subjects limits the generalizability of our results. However, we opted against excluding the female subjects entirely, as a predominantly male population best reflects the reality in forensic psychiatric institutions. 

## 5. Conclusions

Under the challenging circumstances of forensic psychiatry, finding the smallest set of factors that can predict specific risks with reasonable accuracy may help to adequately distribute scarce resources and improve results. Despite its limitations, our findings could demonstrate an example of the use of ML in the development of an easy-to-use predictive model based on few objectifiable factors. Clinicians may decide to allocate resources and interventions accordingly, such as closer monitoring through trained staff or admission to specialized wards. Although the proposed model needs further validation, ideally in a prospective approach with a larger sample, in order to provide generalizability and resilience, it could, in the future, provide institutions with a screening tool for risk stratification and allow the prevention of adverse events, as well as an individualized and more resource-efficient treatment for offender patients with SSD. In addition to more future research, there is a need for a societal and expert community discourse regarding ethical aspects of the implementation and practical application of such models. 

## Figures and Tables

**Table 1:** Sociodemographic data.

Characteristics	Total n/N (%)	No CA/Conviction n/N (%)	Prior CA/Conviction n/N (%)
Male sex	325/356 (91.3)	81/91 (89)	244/265 (92.1)
Age at admission (mean, SD)	34.3 (10.3)	35.1 (11.4)	33.9 (9.9)
Native Country Switzerland	166/356 (46.6)	35/91 (38.5)	131/265 (49.4)
Single (at offence)	286/352 (81.3)	60/88 (68.2)	226/264 (85.6)
Schizophrenia	286/356 (80.3)	70/91 (76.9)	216/265 (81.5)

Note. SD = standard deviation; N = total study population; n = subgroup with characteristics; CA = compulsory admission.

**Table 2:** Machine Learning Models and their performance in nested cross-validation.

Algorithm	Balanced Accuracy (%)	AUC	Sensitivity (%)	Specificity (%)	PPV (%)	NPV (%)
Logistic Regression	66.70	0.73	64.1	69.3	84.3	40.4
Tree	64.90	0.67	65.6	64.1	83.3	40.6
Random Forest	60.7	0.69	76.9	44.5	79.3	42.4
**Gradient** **Boosting**	**69.1**	**0.76**	**68.7**	**69.5**	**86.9**	**45.2**
KNN	60.1	0.66	67.1	53	80	34.1
SVM	63.7	0.71	72.1	55.2	81.3	42.4
Naive Bayes	64.8	0.74	59.6	69.9	85.5	39.5

Note: AUC = area under the curve (level of discrimination); PPV = positive predictive value; NPV = negative predictive value; KNN = k-nearest neighbors; SVM = support vector.

**Table 3:** Absolut and relative distribution of predictor variables most relevant.

Variable Code	Variable Description	No Prior CA/Conviction	Prior CA/Conviction
DZ7	Antisocial behavior	32/89 (36)	130/263 (49.4)
DZ10	Rule breaking	14/88 (15.9)	86/262 (32.8)
PH14b	Cannabis abuse/dependence	36/91 (39.6)	182/265 (68.7)
W3a	Type of Delusion: grandeur	8/81 (9.9)	70/238 (29.4)
R9c	Olanzapine-Equivalent half-life (mean, SD)	17.42 (14.54)	21.21 (14.75)
R14b	Compulsory measure during current forensic hospitalization: isolation	14/88 (15.9)	89/256 (34.8)
PA4	Adopted PANSS at admission: Scale Hyperactivity		
	Symptoms substantially	19/89 (21.3)	70/255 (27.5)
	Symptoms discreetly	13/89 (14.6)	42/255(16.5)
	Symptoms absent	57/89 (64)	143/255 (56.1)
PA17	Adopted PANSS at admission: Scale Guilt feelings		
	Symptoms substantially	14/89 (15.7)	10/255 (3.9)
	Symptoms discreetly	14/89 (15.7)	20/255 (7.8)
	Symptoms absent	61/89 (68.5)	225/255 (88.2)
R20b	Physical violence against staff	5/90 (5.6)	42/265 (15.8)
R28	Estimated future legal prognosis		
	Favorable	27/77 (35.1)	45/227 (19.8)
	Sufficient	26/77 (33.8)	53/227 (22.9)
	Doubtful	12/77 (15.6)	47/227 (20.7)
	Unfavorable	12/77 (15.6)	83/227 (36.6)

Note: SD = Standard deviation; PANSS = positive and negative syndrome scale; CA = compulsory admission. All items reflect the contents of the patients’ files. Due to the retrospective design, a direct evaluation with the patient through the investigators was not possible. For a detailed variable definition and rating procedures for each variable, please refer to the coding document listed in the data availability statement.

**Table 4:** Final gradient boosting model performance measures.

Performance Measures	% (95% CI)
Balanced Accuracy	67.1 (57.3–76.7)
AUC	0.72 (0.62–0.82)
Sensitivity	90.2 (81.2–95.4)
Specificity	50 (28.8–71.2)
PPV	87.1 (77.6–93.1)
NPV	57.8 (33.9–78.9)

Note: AUC = area under the curve (level of discrimination); PPV = positive predictive value; NPV = negative predictive value.

## Data Availability

The dataset generated and analyzed during the current study are available from the corresponding author on reasonable request. A detailed list of all our variables (including definitions and references) is available under the following link: https://www.researchgate.net/publication/363044110_Coding_protocol_Pathways_into_delinquency_in_offenders_suffering_from_schizophrenia_spectrum_disorders (accessed on 28 August 2022).

## Appendix A

**Table A1:** Final hyperparameters for model building during nested cross-validation ^1^.

Algorithm	Hyperparameter
Logistic Regression	does not apply
Tree	minisplit = 1; cp = 0.003; maxcomplete = 4; maxsurrogate = 5; usesurrogate = 2; surrogatestyle = 0; maxdepth = 30;xval = 10
Random Forest	Ntree = 100; replace = true; nodesize = 4; importance = false; localImp = false; mtry = 3
Gradient Boosting	distribution = Bernoulli; n.tress = 2000; cvfolds = 0; interaction.depth = 5; n.minobsinnode = 10; shrinkage = 0.001; bag.fraction = 0.5; train.fraction = 1
KNN	K = 20, distance = 10, integer = 7; numeric = 2; logical = true
SVM	cost = 5; nu = 0.5; kernel = radial; degree = 3; cachesize = 40; tolerance = 0.001; shrinking = true, gamma = 10
Naive Bayes	laplace = 0

^1^ Note: NN = k-nearest neighbors; SVM = support vector machines.
